# Knowledge, attitude and practices (KAP) towards Anthrax among livestock farmers in selected rural areas of Bangladesh

**DOI:** 10.1002/vms3.561

**Published:** 2021-07-07

**Authors:** Pallab Kumar Dutta, Hiranmoy Biswas, Jahir Uddin Ahmed, Md. Shakif‐Ul‐Azam, Bepari Mohammed Jafar Ahammed, Anita Rani Dey

**Affiliations:** ^1^ Department of Livestock Services Dhaka Bangladesh; ^2^ Department of Parasitology Faculty of Veterinary Science Bangladesh Agricultural University Mymensingh Bangladesh; ^3^ Adjunct Faculty American International University—Bangladesh Dhaka Bangladesh

**Keywords:** anthrax, Bangladesh, knowledge, attitude and practice (KAP), Meherpur, Sirajgonj

## Abstract

**Background:**

*Bacillus anthracis* is a zoonotic bacterium that affects wide numbers of vertebrate animals and man and has life threating potential both in animal s as well as humans.

**Methods:**

A cross sectional study was conducted to assess the knowledge about, attitudes towards, and practices addressing (KAPs) anthrax among community members in selected upazillas’ of Meherpur and Sirajgonj districts for the prevention and control of anthrax using a structured questionnaire.

**Results:**

A total of 424 community members were considered in this study irrespective of their age and sex. Most of the respondents were female (57.54%) and about half were illiterate (47.40%). Most of the respondents (86.32%) were self‐employed with crop and livestock farming. Among the self‐employed farmers, cattle (63.73%) were the highest reared animals. Among the respondents, 37.26% had no knowledge about anthrax. On the other hand, among the existing knowledge level, 46.69% received information of anthrax from neighbour, 74.05% and 56.82% were concerned about the mode of transmission of anthrax from animal to human through eating, handling and soil. Respondents usually collected vaccine from quack (58.25%) and vaccination status was highest in Kamarkhand (52.03%) and lowest in Gangni upazilla (10.82%). Overall 62.74% community members considered that anthrax is a fatal disease for livestock and 82.54% people disposed carcass in buried method.

**Conclusion:**

The study findings indicated that the community members had average knowledge on cause, symptoms, transmission and prevention of anthrax. The supplied vaccine was found negligible with the number of livestock in the studied upazilas. Veterinary and Medical health planners should design and implement interventions for awareness building on anthrax under One Health (OH) approach for educating the community people on anthrax control and prevention.

## Graphical abstract

This is the first study to address the perspectives of farmers on anthrax prevention. The study provided basic information on Knowledge, Attitude and Practice on Anthrax. It also helps in designing control strategy on anthrax under One Health (OH) approach.

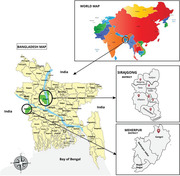

## 1 INTRODUCTION

Anthrax is a zoonotic disease that affects humans, livestock, companion animals and wild‐lives (Fasanella et al., 2010). It is a rare disease in developed countries (Dragon & Rennie, 1995), but still an endemic disease in developing countries (Shivachandra et al., 2016). The causal agent of anthrax, *B. anthracis* establishes a bimodal lifecycle where spore form is developed in the environment and vegetative form within the host. The spores can survive in the environment at adverse condition for several years and initiate infection when favourable conditions are provided (Raymond et al., 2010). The livestock sector is an important sector to improve the livelihood of the majority of rural people in many developing countries (Dey et al., 2020). They provide food, companionship, socio‐cultural activities and are a source of income in various ways as they have an important economic role by sale and services of these animals and their products (Hossain et al., 2015). This dependence on livestock makes people vulnerable to zoonotic diseases. Anthrax is considered as the second prioritized zoonotic disease in Bangladesh . Livestock rearing system plays a vital role in transmitting anthrax from animal to human where there is a close contact between animals and owners. Cattle are mainly affected by anthrax among other herbivores due to its grazing nature. Bovine anthrax outbreak and mortality are four times and thirteen times more than sheep anthrax outbreak and mortality, respectively. Along with animal anthrax, about 20,000–100,000 incidence of human anthrax occur per year globally with significant economic loss (Dutta et al., 2011). Since 1989, human anthrax outbreaks have not been reported in Bangladesh but animal anthrax was reported routinely (International Centre for Diarrhoeal Diseases Research, 2009). In 2009–2010, 14 outbreaks of animal and human anthrax where 140 animal and 273 human cases of anthrax were identified in Pubna, Sirajgong and Tangail district, Bangladesh (A. Chakraborty et al., 2012).Outbreak of animal anthrax in a particular area mostly associated with ecological, demographic, and sociocultural factors (Sitali et al., 2018). Human anthrax is very much linked with animal anthrax. Human anthrax infection has been categorized into Agricultural and Industrial. Human get infection from infected or dead animals accidentally, during slaughtering (agricultural) or from cleaning or processing of infected animal products and by‐products such as contaminated meat, hair, wool, hides and skin (industrial) (Bischof et al., 2007).

Adequate knowledge regarding this disease is essential for early recognition, detection and notification. Inadequate monitoring, surveillance and disease reporting, lack of public awareness, unrestricted movements, poor management and vaccination strategies are the major factors to set an appropriate control strategy against anthrax (Mondal & Yamage, 2014). Bangladesh continues to experience outbreaks of anthrax diseases in livestock despite vaccination as part of a government effort to control them. Among the high‐risk areas of Bangladesh, several outbreaks of anthrax in animals and humans have been reported in Sirajgonj and Meherpur districts during last few years. Epidemic swath of anthrax of 2010, these two districts had marked as ‘Red alert’ according to OIE (Office of International Epizootics, World Animal Health Organization) and WHO (World Health Organization). Since 2010, continuous outbreak of animal anthrax was common in these areas (A. Chakraborty et al., 2012); however, there is lack of information on knowledge, attitudes and practices regarding anthrax among community people, which are fundamental for better understanding about the extent of knowledge among people and for guiding on effective prevention and control measures. Therefore, the study was conducted with the aim of assessing baseline information, knowledge, describe attitudes towards, and determine practices regarding anthrax among community members in selected upazillas’ of Meherpur and Sirajgonj districts for the prevention and control of anthrax on smallholder farms.

## 2 METHODS AND MATERIALS

### 2.1 Ethical statement

Ethical approval was obtained from concerned authority, that is, ERB of AIUB, during data collection. Respondent's dignity and respects were maintained and interviews were taken with strict privacy. There were no potential risks that might cause any harm to study respondent. They were ensured that their personal identity was kept confidential and the data will be used only for study purpose. Moreover, respondents were allowed to withdraw themselves at any stage of the study.

### 2.2 Study design and location

**FIGURE 1:**
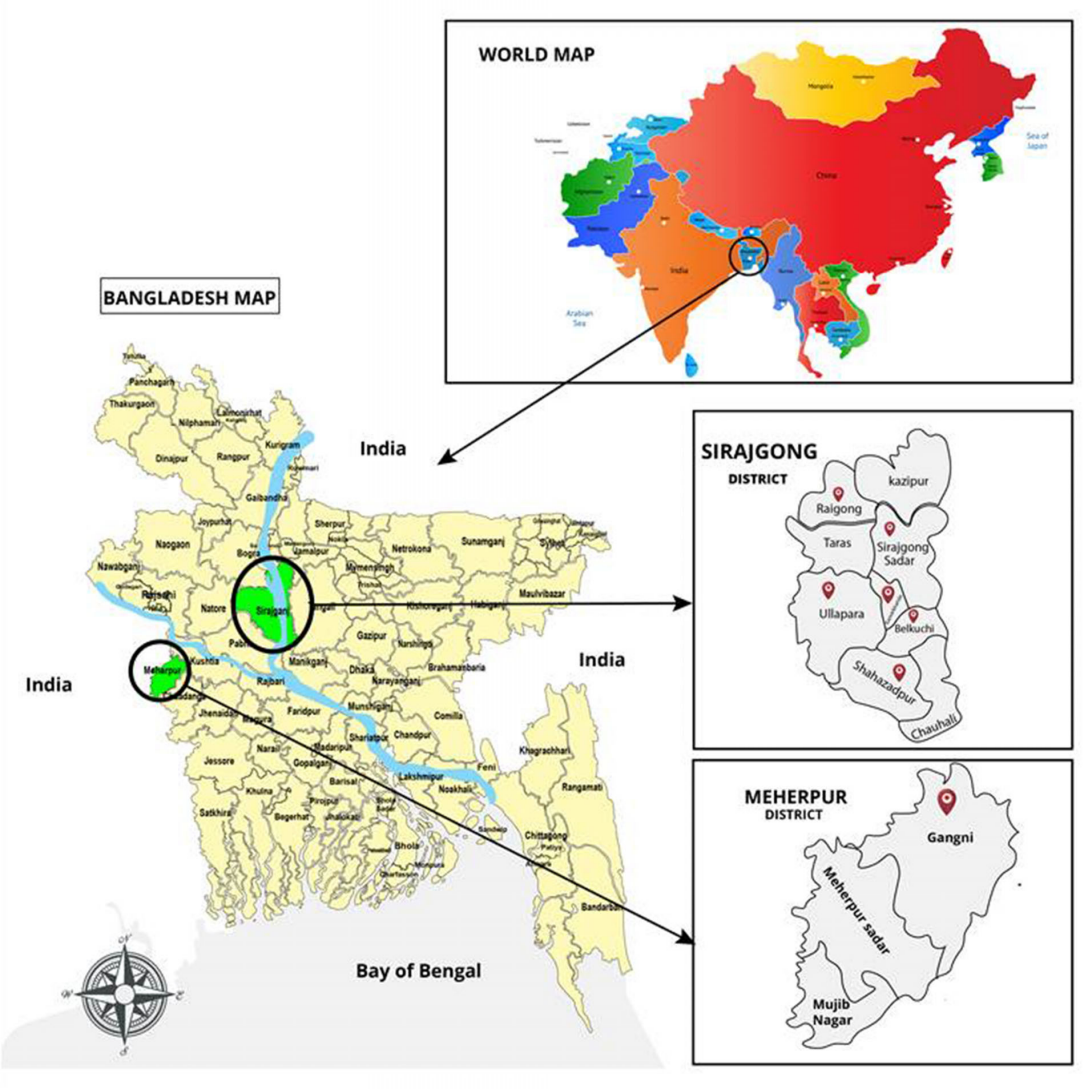
Selected Upazillas of Sirajgonj and Meherepur districts for KAP study

A cross‐sectional descriptive study was conducted in Meherpur and Sirajgonj District for the period of 6 months from September, 2019 to February, 2020. Six upazilla (small administrative area) including Shahajadpur, Ullahpara, Kamarkand, Belkuchi, Raiganj, Sirajgonj sadar from Sirajgonj district and one upazilla including Gangni were considered for this study (Figure 1).

### 2.3 Study population

Purposive sampling was conducted to identify respondents. The purpose of collecting this information was to compare level of knowledge, attitudes and practices among community members who raised livestock. After explaining the purpose of the study and obtaining verbal consent, face‐to‐face interview was taken using the questionnaire. Respondents were provided with a detailed explanation of each question in a language in which they are conversant. They were assured that the supplied information will be kept confidential and will be used only for the study purpose.

### 2.4 Sample size calculation

In order to generate sufficient information of the knowledge, attitudes and practices regarding anthrax among community members in Meherpur and Sirajgonj, sample size was determined by the following formula: *n* = *Z* ^2^ *P*(1 – *P*)/*d* ^2^. Where *n* = required sample size, *Z* = confidence level at 95% (standard value 1.96), *P* = proportion (0.5) is the expected proportion of community members with knowledge on cause/symptom or mode of transmission of anthrax, *d* = level of precision at 5%. *n* = 1.96**2** × 0.5 × 0.5/ (0.05)**2 **= 3.8416 × 0.25/0.0025 = 384.16 ∼ 385. Nonresponse = 10% = 38.5∼39. Then, required sample size was = (385 + 39) = 424.

### 2.5 Development of research instrument

Self‐structured questionnaires were used for data collection from community respondents of the survey areas. The questionnaires captured information on socio‐demographic variables, knowledge, attitudes and practices regarding anthrax. A questionnaire designed for this study was partly adapted from similar studies conducted elsewhere (Bingham, 2010; Matibag et al., 2007). It consisted of 06 closed and 12 open‐ended questions. The questionnaire consisted of three segments: (1) items regarding the respondent and socio‐demographic information (age, sex, education level, occupation, number of people in the household, animal ownership status, location); (2) questions related to the knowledge and perception of anthrax; and (3) questions related to attitudes and perception of anthrax and its control activities. The questionnaire was prior tested in order to improve clarity and interpretation.

### 2.6 Data analysis

At the end of each day of data collection, all questionnaires were handed over and reviewed by the investigator to ensure that all variables had been correctly filled. Data from household questionnaires were entered into Excel spreadsheet, scored and further exported to SPSS for descriptive analysis. Data collected through key informant interviews was analysed using thematic analysis procedures. The data were used to compliment and elaborate quantitative findings and clarify relevant aspects of anthrax related practices and behaviour.

## 3 RESULTS

### 3.1 Socio‐demographic characteristics of participants

The study area covered six upazillas in Shirajganj district and one upazilla in Meherpur district and a total of 424 participants were considered in different upazillas. The socio‐demographic characteristics of the participants in the study area had been represented in the Table 1. All the participants were between 20 and 69 years of age, however, most of them were of middle age (30 and 49 years old—54.94%). The majority participants were female (57.54%). About half of the participants (47.42%) had no formal education and more than half of the participants were literate at different level such as primary level (32.78%), secondary level (8.49%) and tertiary level (11.32%). According to occupation, participants mainly were based on agriculture (86.32%), that is, self‐employed with crop and livestock farming, and only few were involved in household works (10.14%) and business (3.53%). In the study area, farmers predominantly reared cattle (63.73%) as the main source of income to increase their livelihood followed by goats (29.60%), sheep (6.39%) and buffaloes (0.28%).

**TABLE 1:** Socio‐demographic characteristics of participants

Variables	Frequency	Percentage (%)
Upazillas		
Shahjadpur	87	20.51
Kamarkhand	56	13.20
Ullahpara	51	12.02
Sirajgonj sadar	64	15.09
Raigonj	60	14.15
Belkucchi	52	12.26
Gangni	54	12.73
Age		
20–29 years	73	17.21
30–39 years	129	30.42
40–49 years	104	24.52
50–59 years	83	19.57
60–69 years	35	8.25
Gender		
Male	180	42.45
Female	244	57.54
Education level		
Illiterate	201	47.41
Primary education	139	32.78
Secondary education	36	8.49
Tertiary level of education	48	11.32
Occupation		
Agriculture	366	86.32
Housewife	43	10.14
Business	15	3.53
Livestock species		
Cattle	811,957	63.73
Goat	377,081	29.60
Sheep	81,351	6.39
Buffalo	3568	0.28

### 3.2 Knowledge of participants towards anthrax

Responses of participants pertaining to their knowledge towards anthrax had been shown in the Table 2. Majority of the participants (62.73%) had knowledge about anthrax. Among 424 participants, 46.69% participants heard about anthrax from neighbours within the community, 28.30% from local market, 12.97% from upazilla veterinary hospital and 12.02% from quack (untrained people who practiced without permission of legal authority). Majority of the community members (60.14% and 56.60%) could correctly describe one or more symptoms of animal and human anthrax, respectively, while 39.85% and 43.39% did not have any idea about symptoms of animal and human anthrax, respectively. About 47% of participants understood the mode of transmission of anthrax from animal to human through eating, whereas 7.07% and 2.59% of them knew the way of transmission of anthrax through handling of infected meat and contaminated soil, respectively. Rest 43.16% participants had no knowledge on mode of transmission of anthrax from animal to human. Only 39.62% and 8.02% participants had knowledge about the preventive method of anthrax, that is, vaccination and anthrax outbreak, respectively. Majority of the participants (78.3%) had knowledge about the relationship between sudden death of animals and anthrax.

**TABLE 2:** Response of participants pertaining to their knowledge towards anthrax

Variables	Frequency	Percentage (%)
Knowledge about anthrax among respondent		
Yes	266	62.74
No	158	37.26
Source of information about anthrax among respondent		
Neighbour	198	46.69
Local market	120	28.30
Upazila Veterinary Hospital	55	12.97
Quack	51	12.02
Knowledge about symptoms of animal anthrax		
Yes	255	60.14
No	169	39.85
Knowledge about symptoms of human anthrax		
Yes	240	56.61
No	184	43.39
Knowledge on mode of transmission of anthrax from animal to human		
Eating	200	47.16
Handling	30	7.07
Soil	11	2.59
Do not know	183	43.16
Knowledge about preventive method of anthrax (Vaccination)		
Yes	168	39.62
No	256	60.38
Knowledge on anthrax outbreak		
Yes	34	8.02
No	390	91.98
Knowledge about relationship between anthrax and sudden death of animals		
Yes	332	78.3
No	92	21.7

### 3.3 Attitude and practices of respondents towards anthrax

Vaccination is one of the most important preventive measures in any infectious disease. The vaccination status of different upazillas was assessed according to data from register book of Govt. Veterinary Hospital. The highest vaccination status was found in Kamarkhand upazilla (52.03%) followed by Ullahpara (39.80%), Belkuchi (33.33%), Rajgonj (19.49%), Sirajgonj sadar (16.71%), Shahjadpur (12.53%) and Gangni (10.82%). In the study areas, most of the participants (58.25%) collected vaccine from quack followed by Govt. Veterinary Hospital (36.79%) and least of the participants collected vaccine from pharmacy (3.07%) and other source (1.88%). Only one‐third (29.48%) participants were interested to vaccinate their animals against anthrax. Most of the participants (62.74%) considered that anthrax is a fatal disease. Majority of the participants (96.93%) did not participate in affected animal slaughtering. Participants mainly disposed carcass by buried method (82.54%). Very few participants disposed carcass by throwing in water (9.66%), throwing at roadside (4.95%) and allowing cobbler for skinning (2.83%) (Table 3).

**TABLE 3:** Attitude and practices of respondents towards anthrax

Variables	Frequency	Percentage (%)
Attitude		
Vaccination status in different upazilla (dose of anthrax vaccine)		
Shahjadpur (390,938)	49,000	12.53
Ullahpara (170,841)	68,000	39.80
Sirajgonj sadar (185,525)	31,000	16.71
Belkuchi (84,007)	28,000	33.33
Rajgonj (152,929)	29,800	19.49
Kamarkhand (77,063)	40,100	52.03
Gangni (212,654)	23,000	10.82
Source of vaccine		
Quack	247	58.25
Govt. Veterinary hospital	156	36.79
Pharmacy	12	3.07
Others	8	1.88
Vaccination of animals against anthrax		
Yes	125	29.48
No	299	70.52
Anthrax considered as fatal disease		
Yes	266	62.74
No	158	37.26
Practices		
Participation of respondent in affected animal slaughtering		
Not involved in slaughtering	411	96.93
Involved in slaughtering	13	3.07
Carcass management		
Buried	350	82.54
Thrown in water	41	9.66
Thrown at roadside	21	4.95
Allow cobbler for skinning	12	2.83

## 4 DISCUSSION

*B. anthracis* is a Gram‐positive, spore‐forming bacterium and remains an important global public health problem particularly in developing countries. Globally, there are around 1.83 billion people living within anthrax‐risk areas, It may be categorized as emerging, re‐emerging and neglected zoonotic disease (Ngetich, 2019) and a considerable economic loss for the smallholder farmers is associated with reduced performance and sudden death of animals by anthrax (Nayak et al., 2019). Limited general public awareness campaigns have been conducted across the globe.

Anthrax is most common in agricultural regions of Central and South America, sub‐Saharan Africa, Central and Southwestern Asia and Southern and Eastern Europe (Sweeney et al., 2011). Globally, about 63.8 million poor farmers and 1.1 billion livestock were exposed to anthrax infection (Carlson et al., 2019). Among south Asian countries, Bangladesh and India are endemic for anthrax (Nayak et al., 2019). But in this endemic area, anthrax monitoring programs such as diagnosis, case recording and submission of the data to the central authority remain inadequate (Sahoo et al., 2020).

Both human and livestock are vulnerable to anthrax throughout Asia, Africa and North America, especially in arid and tropical rural areas (Carlson et al., 2019). Anthrax transmission is greatly influenced by human behaviour. This behaviour is largely affected by the knowledge, attitudes and practices of the community members. In the studied area, 86.32% participants were fully dependent on agriculture and these rural communities mainly exercise conventional agricultural practice. *B. anthracis* usually presents in the soil and spores can persist for several years in the environment under favourable condition and transmit to animal host through grazing, usually by ingestion or inhalation (Sweeney et al., 2011). Among human population, veterinarians, livestock farmers, any person that handles animal products (such as butchers, wool sorters, tannery workers, etc.), and laboratory personnel are the highest risk group (Shivachandra et al., 2016).

The socio‐cultural practices including slaughtering of sick animals, eating or handling meat from infected animals, and dumping of carcasses in the open have attributed to anthrax transmission in Africa and Southeast Asian countries (Islam et al., 2013). In Bangladesh, due to cultural restriction of consuming dead animals, they slaughtered their animals when the animals are recumbent and involved in selling meat of slaughtered sick animal to recover at least their investment (Ngetich, 2019). Low socioeconomic status and poor education background combined with poor public health infrastructure creates critical conditions that are conducive for zoonotic transmission of anthrax to the human population (Patil, 2010).

Routine vaccination policy is one of the better strategies for prevention and control of anthrax (Kasradze et al., 2018; Mwakapeje et al., 2018; Traxler et al., 2019). The study areas are endemic for anthrax but the vaccination status in different upazillas were not satisfactory. The highest vaccination status was found in Kamarkhand upazilla and the lowest in Gangni upazilla. Deficient coverage of anthrax vaccine in livestock contributed to the outbreaks animal anthrax. In Bangladesh, livestock anthrax vaccine is supplied by The Livestock Research Institute of the Government of Bangladesh but it is much less than the demand (A. Chakraborty et al., 2012). Government should take strategy of increasing the availability of anthrax vaccine either through increasing domestic production or by importing. Furthermore, to increase the demand of vaccine at farmer level, awareness should build up among the animal owners by considering the economic impact of animal and human anthrax.

In this survey, about 47.41% participants were illiterate. Due to illiteracy, livestock farmers hiding the truth, especially where they were affected negatively or involved with inappropriate activities like slaughtering of sick animal and subsequent selling and consumption of meat from a suspected carcass or even did not report the cases to the relevant authority. Poor public health infrastructure in endemic districts exposes areas to higher anthrax risk (Bhattacharya & Pati, 2019; Patil, 2010; Pérez‐Tanoira et al., 2017; Vaismoradi et al., 2013). However, proper, timely and efficient farmers’ reporting of animals’ sudden deaths, alongside accurate and robust diagnosis of anthrax put in place by the Veterinary services increased confirmed anthrax cases than expected in the rural communities. These findings are consistent with the previous findings of the researchers (Shabani et al., 2015) regarding KAP on Rift Valley Fever (RVF), which outlined that most participants were not sincere where they were affected negatively, and also where the outbreak was highly publicized with high morbidities and mortalities in both humans and animals.

Disposal of the carcass of animals is a source of concern for anthrax transmission. The preferred method of disposal of anthrax infected animal is incineration/buried in the most of the developing countries. In Bangladesh, social–cultural activities do not allow to burn carcass. In this study, 82.54% participants practice buried method but rest of the participants disposed carcass by throwing in water/roadsides and allowing cobbler for skinning. Carnivores and birds play a vital role to drag contaminated meat over the areas, thus, increasing ground contamination with anthrax spore. Dog itself is resistant to anthrax but acts as a mechanical vectors from field to household (Turnbull, 1998).

Proper and early diagnosis is one of the important components for treatment, prevention and control of anthrax. But diagnostic facilities were insufficient in the endemic districts, which is similar with the observations from other studies in Asia and Africa (P. P. Chakraborty et al., 2012; Nayak et al., 2019; Vaismoradi et al., 2013). It was revealed that timely diagnosis can control the outbreak of anthrax (Sahoo et al., 2020).

## 5 CONCLUSIONS

To the best of our knowledge, this is the first study to address the perspectives of smallholder farmers regarding anthrax in Bangladesh. The study revealed that majority of the community members had idea about anthrax and its symptoms in animals and humans. But about half of the participants did not know the mode of transmission of anthrax. The vaccination status was below 50% within the studied area except Kamarkhand (52.04%) and also most of the farmers did not show interest to vaccinate their animals. Participants showed positive attitude in affected animal slaughtering and carcass management. It is necessary to ensure increased public awareness on vaccination of the livestock population along with sufficient coverage of the anthrax vaccine that will make a large contribution to the control of anthrax outbreaks.

## AUTHOR CONTRIBUTIONS

Pallab Dutta: Formal analysis, Investigation, Methodology, Software, Writing‐original draft, Writing‐review & editing. Hiranmoy Biswas: Formal analysis, Methodology, Software, Writing‐original draft, Writing‐review & editing. Jahir Ahmed: Data curation, Investigation, Methodology, Software, Visualization, Writing‐review & editing. Md. Azam: Formal analysis, Investigation, Validation, Visualization, Writing‐original draft, Writing‐review & editing. Bepari Ahammed: Formal analysis, Investigation, Methodology, Software, Writing‐original draft, Writing‐review & editing. Anita Dey: Conceptualization, Data curation, Formal analysis, Funding acquisition, Methodology, Project administration, Supervision, Writing‐review & editing.

## CONFLICT OF INTEREST

The authors have no conflicts of interest to declare.

### PEER REVIEW

The peer review history for this article is available at https://publons.com/publon/10.1002/vms3.561.
